# Chemotherapy-related cognitive impairment as a crisis of the self: a Winnicottian psycho-oncological perspective

**DOI:** 10.3389/fpsyg.2026.1811745

**Published:** 2026-05-14

**Authors:** Sun Mie Kang, Jin Su Kim

**Affiliations:** 1 Department of Literature and Arts Psychotherapy, Graduate School, Konkuk University, Seoul, Republic of Korea; 2 Division of Applied RI, Korea Institute of Radiological and Medical Sciences (KIRAMS), Seoul, Republic of Korea; 3 Radiological and Medical Sciences, University of Science and Technology (UST), Seoul, Republic of Korea

**Keywords:** cancer survivors (CSs), chemotherapy-related cognitive impairment (CRCI), cognition disorders, identity, object relations theory, psycho-oncology, self concept, Winnicottian theory

## Abstract

**Objective:**

Chemotherapy-related cognitive impairment (CRCI) is typically conceptualized as a neurocognitive side effect of cancer treatment, with emotional distress framed as a secondary or reactive consequence. This paper aimed to reconceptualize CRCI from a psycho-oncological perspective by examining how acquired cognitive unreliability disrupts self-experience, identity, and continuity of being, using Donald W. Winnicott’s theory of the self and environment.

**Methods:**

This theoretical paper synthesized Winnicottian concepts—including the True Self, False Self, holding, and play—to develop a conceptual model of CRCI that links cognitive dysfunction to disruptions in selfhood and relational safety.

**Results:**

CRCI is conceptualized as a potential disruption of the continuity of the True Self in a subset of survivors, particularly those whose premorbid identity was strongly organized around cognitive functioning. Within this framework, cognitive unreliability is interpreted as threatening self-continuity, with adaptive responses taking the form of intensified False Self functioning, including vigilance, over-adaptation, and concealment. While these strategies may preserve external functioning, they constrain spontaneity, vitality, and the capacity for play. Recovery is therefore reframed not as cognitive restoration alone, but as the re-establishment of a holding environment that supports the gradual re-emergence of authentic self-experience.

**Conclusion:**

Within this framework, addressing CRCI is not limited to cognitive remediation, but extends to the relational conditions that support the continuity of the self and the possibility of authentic lived experience in a subset of survivors.

## 1 Introduction

Chemotherapy-related cognitive impairment (CRCI), or chemobrain, refers to persistent cognitive difficulties affecting memory, attention, processing speed, learning, and executive function in a substantial proportion of cancer survivors, with epidemiological studies estimating a prevalence of 35–75% (Chapman et al., 2022; Oppegaard et al., 2024). Within this dominant framework, cognitive change is treated primarily as a functional impairment, while accompanying emotional distress is typically regarded as a secondary or reactive consequence (Sturm et al., 2013).

However, accumulating qualitative and psycho-oncological research indicates that such accounts fail to capture the core of many survivors’ experiences. For a substantial proportion of individuals, CRCI is not merely cognitive inefficiency but a profound disruption in how the self is constituted, trusted, and lived over time, particularly in professional and relational contexts (Ander et al., 2018).

Survivors frequently describe cognitive unreliability as a rupture in personal continuity, often expressed as feeling that “a part of me is gone” or that they are “no longer who they used to be.” Such accounts point to an ontological injury-a disturbance in the implicit sense of being a coherent and reliable subject. Qualitative studies, including those describing life with a “new normal,” show that survivors experience themselves as less competent, less intelligent, and fundamentally altered (Henderson et al., 2019; Dehghan et al., 2022). These experiences are enacted through shame, concealment of cognitive lapses, withdrawal from social and occupational roles, and heightened self-monitoring, indicating that cognitive impairment reshapes agency and self-evaluation rather than producing distress only as a downstream effect (Myklebost et al., 2025).

Although anxiety, depressed mood, and reduced self-efficacy are consistently intertwined with everyday cognitive failures in CRCI (West et al., 2022), prevailing psycho-oncological models continue to operationalize distress as an additive or mediating variable (Pang et al., 2023). This approach implicitly frames emotional suffering as reactive rather than as a constitutive dimension of living with cognitive unreliability, leaving the phenomenology of selfhood—how cognitive failure undermines self-trust, identity, and relational confidence—under-theorized (Haywood et al., 2024). Clinically, this gap contributes to interventions for cancer-related cognitive impairment that focus on cognitive rehabilitation or mood symptoms, while neglecting the reconstruction of a coherent and trustworthy sense of self (Henderson et al., 2019; Spinetti and Orzelleca, 2025). Phenomenological accounts conceptualize illness not merely as functional impairment, but as a disruption of one’s being-in-the-world and the continuity of self-experience (Carel, 2018). However, while phenomenology powerfully describes the experiential rupture induced by illness, it offers limited resources for understanding how such disruptions are relationally organized, defended against, and potentially restored over time.

To address this gap, the present paper draws on the psychoanalytic theory of Donald W. Winnicott. His developmental model of the self–environment relationship, particularly the distinction between the True Self and the False Self, provides a conceptual vocabulary for understanding psychic disruption under conditions of bodily threat and environmental unreliability. The True Self refers to spontaneity, aliveness, and continuity of being, whereas the False Self emerges as a defensive organization when spontaneous expression must be replaced by compliance and control (Winnicott, 1953; Winnicott, 1954). This Winnicottian theory of the True Self and the False Self closely parallels the lived experience of CRCI. Faced with internal cognitive unreliability and persistent external expectations of competence, many survivors—especially those whose identity has been closely tied to cognitive functioning—adopt intensified strategies of vigilance and self-monitoring. While these strategies may preserve outward performance, they often do so at the cost of exhaustion, emotional constriction, and alienation from one’s own vitality (Evans et al., 2016). The present paper proposes a conceptual reconceptualization of CRCI grounded in Donald W. Winnicott’s theory of the self and environment. Rather than treating cognitive impairment as a discrete functional deficit with secondary emotional consequences, this framework situates cognitive unreliability within the continuity of self-experience and relational safety. Importantly, this model is explicitly intended for a subset of CRCI survivors for whom premorbid cognitive functioning constituted a central axis of self-definition, and is not proposed as a general model of CRCI. Table 1 provides a conceptual comparison between conventional models of cancer-related cognitive impairment and a Winnicottian framework across key levels of analysis.

**Table 1:** Conceptual comparison of conventional and Winnicottian models of cancer-related cognitive impairment.

Level	Conventional CRCI model	Winnicottian framework (This Study)
Biological	Neuroinflammation; large-scale network dysfunction	Biological disruption as a background condition influencing self–environment regulation
Cognitive	Deficits in memory, attention, and executive function	Cognitive unreliability experienced as a threat to self-continuity
Psychological	Emotional distress conceptualized as a secondary or reactive response	Emotional distress conceptualized as constitutive of the disruption
Self / Identity	Largely unaddressed or treated implicitly	Crisis of the True Self and disruption of continuity of being
Clinical Focus	Cognitive remediation and compensatory strategies	Holding, and the restoration of play and livability

## 2 Theoretical and conceptual framework

To situate this psychoanalytic reconceptualization within existing scholarship, the following section reviews current neurocognitive, biological, and psycho-oncological models of CRCI. By examining their empirical contributions and conceptual limits, the review highlights the need for an integrative framework capable of addressing not only cognitive dysfunction but also the disruption of selfhood and lived experience characteristic of CRCI.

### 2.1 Neurocognitive and biological perspectives

#### 2.1.1 Neurocognitive profile

From a neuropsychological standpoint, CRCI is generally described as a mild to moderate decline across several core cognitive domains, rather than a global dementia-like syndrome (Wefel et al., 2015). A recent systematic review and meta-analysis in breast cancer populations demonstrated significant post-chemotherapy declines in verbal and visual memory, psychomotor speed, and sustained or divided attention (Godaert and Drame, 2025).

#### 2.1.2 Neuroimaging evidence

Positron emission tomography (PET) and Magnetic Resonance Imaging (MRI) studies indicate that metabolic hypofunction and compensatory hyperactivation, together with structural and network-level disruptions in frontal, cingulate, and large-scale cognitive networks, contribute to deficits in attention, memory, and executive function (Ahles and Root, 2018; Kesler et al., 2013; Sorokin et al., 2014; Lim et al., 2016; Lee et al., 2019). Recent neuroimaging studies increasingly frame CRCI as a disorder of large-scale brain network dysfunction rather than focal structural injury, reporting altered connectivity and activity within the default mode, central executive, and dorsal attention networks across cancer survivor cohorts (Silverman et al., 2025).

#### 2.1.3 Molecular and cellular mechanisms

Many chemotherapeutic agents, including alkylating agents, platinum-based drugs, and topoisomerase inhibitors, induce DNA crosslinks and strand breaks in neural progenitor cells and mature neurons, overwhelming DNA repair pathways and promoting neuronal dysfunction or cell death. In parallel, oxidative stress and mitochondrial dysfunction play a central role in neurotoxicity. Agents such as cisplatin, doxorubicin, and methotrexate increase reactive oxygen species, damage mitochondrial DNA, and impair oxidative phosphorylation, leading to ATP depletion and activation of neuronal apoptotic pathways (Kim et al., 2025). Chemotherapy also induces persistent neuroinflammation through elevation of peripheral and central pro-inflammatory cytokines, including TNF-*α*, IL-1β, and IL-6. These cytokines activate microglia and astrocytes, disrupting synaptic plasticity and neuronal homeostasis (Chen et al., 2021). In experimental models of cisplatin- and doxorubicin-induced neurotoxicity, cognitive deficits are closely associated with microglial activation, NF-κB signaling, and upregulation of inflammatory mediators (Mostafa et al., 2025). At the synaptic level, chemotherapy alters dendritic spine density and morphology in hippocampal and cortical neurons, resulting in impaired long-term potentiation and synaptic signaling (Fahim et al., 2019). Additionally, brain-derived neurotrophic factor (BDNF) levels in the hippocampus and serum are reduced following exposure to agents such as 5-fluorouracil, doxorubicin, and methotrexate, and these reductions are associated with worse cognitive performance in both patients and animal models (Nguyen and Ehrlich, 2020).

#### 2.1.4 Clinical implications

Standardized consensus definitions and cognitive assessment batteries are essential to harmonize clinical trials and enable guideline-level recommendations (Alhowail, 2025). Precision risk stratification incorporating host factors, treatment exposures, comorbidities, and molecular or transcriptomic biomarkers of neuroinflammation and mitochondrial dysfunction is increasingly critical for individualized clinical management (Acharya et al., 2025). In parallel, scalable digital and remote cognitive rehabilitation platforms, often integrating physical exercise and mindfulness components, represent a sustainable and promising strategy for survivorship care interventions (Rovito et al., 2025).

### 2.2 Psycho-oncological and psychosocial perspectives

Within psycho-oncology, CRCI is recognized as a key determinant of psychological adjustment and survivorship quality of life. CRCI is associated with anxiety, depression, fatigue, reduced self-efficacy, social withdrawal, and occupational distress, with depression partially mediating the relationship between objective cognitive function and quality of life. Accordingly, psycho-oncological care increasingly integrates routine screening for psychological distress, mood disturbances, and subjective cognitive complaints into cancer care (Oh and Kim, 2016). Qualitative studies describe CRCI as a disruption of identity and adaptation to a “new normal,” characterized by reduced confidence in work performance and social roles, even when objective cognitive changes are modest. Survivors frequently report frustration, perceived stigma, fear of job loss, and interpersonal strain related to the invisibility of cognitive symptoms (Galica et al., 2012). Subjective cognitive complaints are strongly influenced by psychological distress, sleep disturbance, fatigue, stress, and lifestyle factors such as physical inactivity, independent of neuropsychological test performance (West et al., 2022). Coping style, illness beliefs, and social support further shape whether CRCI is perceived as a manageable treatment-related effect or as a marker of permanent cognitive decline (Rovito et al., 2025). To further situate this framework within the broader psycho-oncological literature, it is important to consider its relation to other established interpretive approaches, including cognitive-behavioral, trauma-informed, narrative identity, phenomenological, self-discrepancy, and stress-appraisal models.

#### 2.2.1 Competing interpretive frameworks

The Winnicottian reconceptualization of chemotherapy-related cognitive impairment (CRCI) as a crisis of the True Self sits alongside, and in tension with, established frameworks in psycho-oncology and health psychology. Rather than replacing these perspectives, the model functions as a depth-oriented supplement highlighting dimensions of self-experience, holding, and play that are under-described in existing accounts.

#### 2.2.2 Cognitive behavioral and self discrepancy approaches

Cognitive behavioral models conceptualize CRCI-related distress in terms of maladaptive beliefs about competence and prognosis, avoidance patterns, safety behaviors, and reduced self-efficacy, drawing on broader CBT formulations of cancer adjustment. Work in oncology emphasizes how negative automatic thoughts, catastrophizing, and rigid performance standards contribute to anxiety, depression, and functional impairment. Lazarus and Folkman’s transactional model describes how appraisals and coping resources shape adjustment under chronic threat. Self-discrepancy theory further explains suffering as a divergence between the “actual” self and internalized “ideal” or “ought” standards, particularly in individuals whose identity was organized around cognitive vitality. Empirical studies in breast cancer survivors show distress, perceived decline, and attempts at reappraisal consistent with these models (Murphy et al., 2021; Osborn et al., 2006; Higgins, 1987). Interventions emphasize cognitive restructuring, graded exposure, and more flexible self-standards. A Winnicottian reading does not reject these mechanisms but situates them within a broader organization of the self: hypermonitoring, rigid demands, and perfectionism are interpreted as intensifications of False Self functioning preserving performance at the cost of spontaneity. Rather than asking whether cognitions are distorted, this framework examines how they operate within a defensive economy maintaining self-coherence.

#### 2.2.3 Trauma informed and stress appraisal models

Trauma-informed perspectives frame cancer and its treatment as potentially traumatic, emphasizing hyperarousal, intrusive recollections, and altered threat perception and bodily trust (Davidson et al., 2023). Reviews of cancer-related PTSD describe clinically significant re-experiencing, avoidance, and hypervigilance, suggesting cancer functions as a prolonged stressor (Buciuc et al., 2025). Recent work links these responses to ongoing threats, bodily unpredictability, and chronic surveillance, situating cognitive complaints within a broader threat-based context. Stress appraisal models similarly conceptualize CRCI as a stressor shaped by threat/loss appraisals and perceived coping resources (Hulbert-Williams et al., 2013; Poreba-Chabros et al., 2022). Distress is associated with vigilance, catastrophic interpretations of lapses, and sustained arousal, which may exacerbate cognitive complaints and fatigue (Bitan et al., 2025). The Winnicottian model converges in recognizing threat and vigilance but shifts focus from symptom clusters to self-organization under unreliable conditions. Hypervigilance is reframed as contraction of potential space and an attempt to sustain continuity of being when body and environment feel unstable.

#### 2.2.4 Phenomenological and narrative identity perspectives

Phenomenological approaches describe illness as a disruption of being-in-the-world, temporality, and bodily orientation, including alienation from one’s body and loss of familiar world structures (Toombs, 1988). These accounts resonate with CRCI survivors’ experiences of feeling slowed, muted, or no longer themselves. Sociological perspectives describe chronic illness as “biographical disruption,” requiring reconstruction of identity and social roles. Narrative identity frameworks extend this by framing survivorship as re-authoring one’s life story, integrating illness into a coherent narrative (Bury, 1982; Williams, 2021). While these perspectives emphasize lived experience, self-continuity, and meaning-making, they often remain agnostic about defensive and relational organizations shaping experience. A Winnicottian model offers a psychodynamic elaboration: disruptions in coherence are linked to intensified False Self adaptation, constriction of play, and failures of holding, while recovery involves restoration of potential space for new narratives.

#### 2.2.5 Points of divergence and complementarity

These frameworks provide overlapping but distinct lenses on CRCI. Cognitive behavioral, self-discrepancy, and stress appraisal models offer operationalizable mechanisms and intervention targets supported by empirical literature (Hofmann et al., 2012; Higgins, 1987). However, they may underrepresent the depth of ontological disruption experienced as a threat to continuity of being. Trauma-informed, phenomenological, and narrative approaches capture experiential and existential dimensions but provide limited guidance on how defensive adaptations are organized and transformed in relational contexts (Cloud et al., 2026; Dag et al., 2026; Tamura et al., 2026; Uzun et al., 2026). The Winnicottian framework is not proposed as a superior account but as a depth psychological lens most applicable in cases of high premorbid cognitive centrality, marked disruption of self-continuity, and dominant False Self functioning. In more functional or transient distress, cognitive or stress-based models may suffice. Clarifying these applicability boundaries is essential to prevent overextension and to support integrative research combining Winnicottian constructs with cognitive, trauma-related, and narrative variables.

### 2.3 Winnicott’s theory in psychosomatic and medical contexts

Although Winnicott’s work is commonly associated with early mother–infant interactions (Winnicott, 1962), Winnicott’s theoretical and clinical implications may extend to contexts of medical illness and psychological crisis. Within psycho-oncology, cancer is increasingly understood as a condition that challenges psychological integrity and self-experience, necessitating forms of care that extend beyond symptom management to encompass emotional containment and relational support (Grassi, 2020). This emphasis resonates with Winnicott’s concept of the holding environment, understood here as the relational conditions that enable patients to endure vulnerability without defensive over-adaptation. Winnicott conceptualized psychosomatic integration as a developmental achievement through which the psyche comes to “inhabit” the body. Disruptions to this process may result in bodily symptoms, dissociation, fragmentation, or the emergence of defensive omnipotence, particularly under conditions of environmental failure. In medical settings, illness can therefore be understood not merely as a biological event but as a situation that exposes the organization of the self and its dependence on environmental reliability. Winnicottian concepts such as the holding environment (Winnicott, 1960b), True and False Self (Winnicott, 1960a), and play (Winnicott, 1968) have increasingly been applied to contexts of chronic illness, surgery, and cancer care (Xie et al., 2023). Clinical accounts suggest that when adequate relational holding is available, medical crises may become opportunities for renewed psychosomatic integration rather than sites of irreversible loss (Xie et al., 2023). However, to date, applications of Winnicott’s framework to CRCI have been very limited. Existing literature typically addresses biological, cognitive, and psychosocial dimensions in parallel, without examining how cognitive disruption may fracture the continuity of the *True Self*, intensify reliance on *False Self* functioning, or constrict the capacity for play, creativity, and spontaneous engagement with life among cancer survivors. A Winnicottian perspective thus provides a novel conceptual framework for understanding CRCI not merely as a cognitive impairment, but as a disruption of psychosomatic continuity and the experience of the self. Although the Winnicottian constructs employed in this framework are theoretical in origin, they need not remain purely abstract. Table 2 integrates key Winnicottian constructs into clinically observable and interpretive dimensions relevant to cancer-related cognitive impairment. These dimensions are not proposed as direct psychometric entities, but as relational and experiential markers that can be approached through qualitative, narrative, and clinical observation.

**Table 2:** Winnicottian constructs as clinically interpretable dimensions of cancer-related cognitive impairment.

Winnicottian construct	Clinical meaning	Experiential indicators	Distinguishing feature	Interpretive focus	Clinical implications
Holding	A relational configuration that provides sufficient environmental reliability for the patient’s experience to unfold without pressure toward immediate coherence, integration, or logical consistency	Disorganized or fragmented narratives; sustained pauses; tolerance of ambiguity; persistence of affect or confusion in a relational context that does not rush toward clarification or repair	Characterized by reduced defensive self-organization in the presence of cognitive disruption, without concurrent pressures toward reassurance, normalization, or narrative repair	Not equivalent to empathy, alliance, or communication quality: holding refers specifically to a receptive stance that suspends demands for clarity or coherence, even when alliance and support are present	Maintain a non-intrusive, receptive stance; avoid premature clarification, reassurance, or interpretation; allow incoherence and temporal extension of experience without corrective pressure
Play	A transitional, symbolic space enabling creative exploration of inner reality, including paradoxical or symbolically impossible configurations, without being organized around problem-solving or symptom reduction	Use of metaphor, humor, or as-if language; exploration of contradictory or unrealistic scenarios; flexible shifting between perspectives; non-goal-directed elaboration that does not aim at advice seeking, reassurance, or solution-testing	Activity is not organized around performance, error avoidance, or self-evaluation; symbolic elaboration is valued in its own right rather than as a means to restore competence or clarity	Not reducible to mood improvement, coping, or problem-solving: symbolic exploration that tolerates impossibility and ambiguity is essential; interpretive closure or premature meaning-making tends to collapse play	Preserve symbolic and exploratory space; avoid translating material into immediate meaning or advice; support creative variation and non-linear exploration, while recognizing that these indicators may overlap with curiosity and mood recovery
Integration	A non-linear, partial process through which previously disorganized experiences may become narratively linkable to one another	Gradual linking of fragmented experiences; capacity to reference prior confusion; coexistence of coherence and discontinuity; evolving but incomplete narratives	Coherence emerges without suppressing contradiction or enforcing resolution, and partial or unstable integration may remain a viable and livable state for individuals with ongoing cognitive disruption	Not equivalent to forced coherence: overly tidy or immediate narrative organization may reflect defensive structuring rather than integration; non-integration is not inherently pathological	Support gradual and tolerable narrative linking; revisit prior disorganization without forcing synthesis; recognize that non-integration may remain a valid and livable state
False Self functioning	A defensive organization that restores apparent coherence and adaptive performance at the cost of spontaneity and authentic experience	Rapid restoration of coherence after disruption; over-articulation; intellectualization; heightened self-monitoring; compliant or performative tone	Coherence is rapidly re-established through over-control rather than emerging from tolerance of disruption.	Not equivalent to healthy regulation: immediate coherence following disruption may indicate defensive compliance rather than genuine integration	Decelerate performance tendencies; avoid reinforcing adaptive façade; create conditions that permit non-performance and experiential authenticity

#### 2.3.1 Reorganizing self-experience in CRCI: a Winnicottian interpretation of survivor accounts

This section illustrates how the proposed Winnicottian framework can be used to interpret recurring patterns described in qualitative studies and survivor accounts of CRCI, rather than introducing new theoretical constructs. Drawing on prior qualitative research, the focus here is on how changes in cognitive reliability are lived and organized at the level of self-experience. Supplementary Table S1 provides illustrative re-readings of conventional CRCI descriptions through a Winnicottian lens.

Across qualitative CRCI studies, survivors whose premorbid sense of self was strongly grounded in cognitive fluency, emotional resonance, and mental vitality frequently describe a qualitatively altered experience of cognitive life following chemotherapy (Sharma and Brunet, 2023). Rather than emphasizing discrete cognitive failures alone, survivors often report a pervasive sense that their thinking has become “muted” or flattened, marked by diminished emotional resonance and reduced experiential vitality. Notably, these descriptions are frequently reported even when basic cognitive operations remain largely intact on formal testing.

From a Winnicottian perspective, such accounts can be read as reflecting a shift toward compliance-dominated modes of self-organization, in which spontaneity and aliveness are constrained in favor of control and predictability. Survivors may describe themselves as continuing to function adequately in daily life while simultaneously experiencing a sense of operating without feeling fully alive, echoing Winnicott’s characterization of False Self-dominated functioning. Importantly, this interpretation does not imply psychopathology, but highlights how defensive adaptations may shape lived experience under conditions of internal unreliability.

A related pattern evident in survivor narratives involves the progressive organization of everyday life around excessive preparation, checking, and error prevention. Individuals whose premorbid identity emphasized competence and reliability often report increasing reliance on external scaffolding—such as lists, routines, and repeated verification—to manage persistent uncertainty about their cognitive performance. Over time, these strategies may shift from situational aids to a dominant mode of navigating daily life.

Within a Winnicottian reading, such patterns may be understood as an intensification of vigilance and self-surveillance aimed at preserving external functioning and social credibility. While often effective in maintaining performance, these adaptations are frequently described as exhausting and emotionally constricting, accompanied by a diminished sense of flexibility, play, and experiential vitality. Survivors may report feeling increasingly estranged from their own lived experience, as everyday activity becomes oriented toward error prevention rather than engagement or enjoyment.

Taken together, these recurring experiential patterns suggest that CRCI may involve not only cognitive inefficiency but a reorganization of how the self is lived and sustained over time. The following section builds on these illustrative observations to articulate a conceptual model of CRCI as a disruption in the continuity of the self.

#### 2.3.2 Toward a conceptual model: CRCI as a crisis of the true self

This framework is explicitly intended for a subset of CRCI survivors for whom premorbid cognitive functioning constituted a central axis of self-definition. From this Winnicottian perspective, CRCI may be conceptualized not merely as a cognitive impairment, but as a crisis in the continuity of the True Self precipitated by acquired cognitive unreliability, consistent with phenomenological accounts of illness as a disruption of being and self-continuity (Carel, 2018). This paper advances a conceptual model in which CRCI is conceptualized as a developmental–relational crisis, defined as a disruption unfolding across cognitive function, relational expectation, and the continuity of self-experience over time. The notion of a “crisis of the True Self” is employed not as a diagnostic category in Winnicott’s original clinical sense, but as a theoretical construct intended to characterize disruptions in the continuity of being arising under conditions of acquired cognitive unreliability. The proposed conceptual model of CRCI, which reframes cognitive impairment as a disturbance in self-experience and self-organization, is summarized in Table 2.

The present framework highlights three interrelated conceptual propositions. First, CRCI directly threatens the continuity of the True Self. For many individuals, cognitive capacities function as the primary medium through which spontaneity, competence, and self-coherence are experienced. When these capacities become unreliable, the individual confronts not only cognitive failure but a destabilization of the implicit sense of being. Neuroimaging findings, including PET-based metabolic and inflammatory alterations, are not treated here as direct explanations of subjective experience, but as a complementary level of description that may reflect disruptions in self–environment regulation accompanying CRCI. Second, in response to cognitive unreliability, survivors often adopt heightened self-monitoring, over-preparation, masking of cognitive lapses, and avoidance of situations that may expose vulnerability. Qualitative studies in psycho-oncology describe these strategies as common efforts to maintain occupational competence, social credibility, and a sense of normality in the face of invisible cognitive symptoms (Henderson et al., 2019; Myklebost et al., 2025). Such sustained vigilance and compensatory control are associated with self-regulatory fatigue, emotional exhaustion, and diminished subjective vitality, particularly when adaptive efforts must be continuously maintained over time (Evans et al., 2016). From a Winnicottian perspective, these patterns can be understood as an intensification of False Self functioning—an initially adaptive mode of organization oriented toward compliance and error prevention, which becomes increasingly constraining over time, leading to exhaustion and alienation from spontaneity and play. As such, the increasing dominance of False Self functioning has direct implications for the erosion of play and creative engagement, forming the third component of the present model. Accordingly, as False Self adaptation becomes dominant, the potential space of play and creativity contracts. Activities that once allowed for experimentation, pleasure, and self-expression are replaced by performance-oriented strategies aimed at error prevention. Over time, this foreclosure of play reinforces the sense that the “real self” has been lost.

From this vantage point, recovery from CRCI cannot be reduced to cognitive remediation alone; rather, it requires the re-establishment of a holding environment in which cognitive limitations, emotional ambivalence, and altered identity can be tolerated without psychological collapse. Within this context, the restoration of play emerges as a central therapeutic task. Importantly, this model is most applicable to a subset of survivors for whom cognitive functioning played a central role in self-definition prior to illness, and for whom cognitive disruption therefore carries particular implications for identity and self-coherence. Although the present paper is theoretical in nature, the Winnicottian constructs employed here—True Self, False Self, holding, and play—need not remain purely abstract or metaphorical. Rather, they can be approached as clinically observable dimensions that are amenable to indirect assessment through triangulation of narrative, relational, and functional measures.

The True/False Self dynamic may be most meaningfully accessed through qualitative and narrative methodologies. Semi-structured interviews focusing on discontinuities between the “previous self” and the “current self” can elucidate disruptions in self-continuity that are not captured by standard neurocognitive testing. In addition, existing psychometric instruments may serve as useful proxies rather than direct measures. For example, the Self-Concept Clarity Scale can reflect the degree to which one’s sense of self is experienced as coherent and stable, while indices of narrative identity coherence—including temporal integration, causal meaning, and thematic continuity—can capture the extent to which individuals are able to integrate cognitive changes into a livable self-narrative.

The concept of holding is primarily relational and contextual, and thus lend themselves to assessment through measures of perceived interpersonal safety and attunement. Therapeutic alliance scales, perceived validation, and clinician responsiveness may serve as indirect indicators of holding, reflecting sensitivity to vulnerability, appropriate pacing of information, and acknowledgment of patients’ cognitive and emotional limitations. Importantly, these dimensions are observable not only within psychotherapeutic settings but also in routine oncological follow-up, where subtle failures of holding may exacerbate experiences of self-fragmentation.

Finally, play, as conceptualized by Winnicott, can be examined through functional and phenomenological indicators rather than direct measurement. Cognitive flexibility, symbolic capacity, imagination, and future-oriented thinking may serve as observable correlates of restored playfulness. Clinically, the re-emergence of play may be reflected in a survivor’s ability to experiment with new identities, tolerate ambiguity, and engage creatively with a changed cognitive landscape, even in the absence of full cognitive recovery.

**Figure 1:**
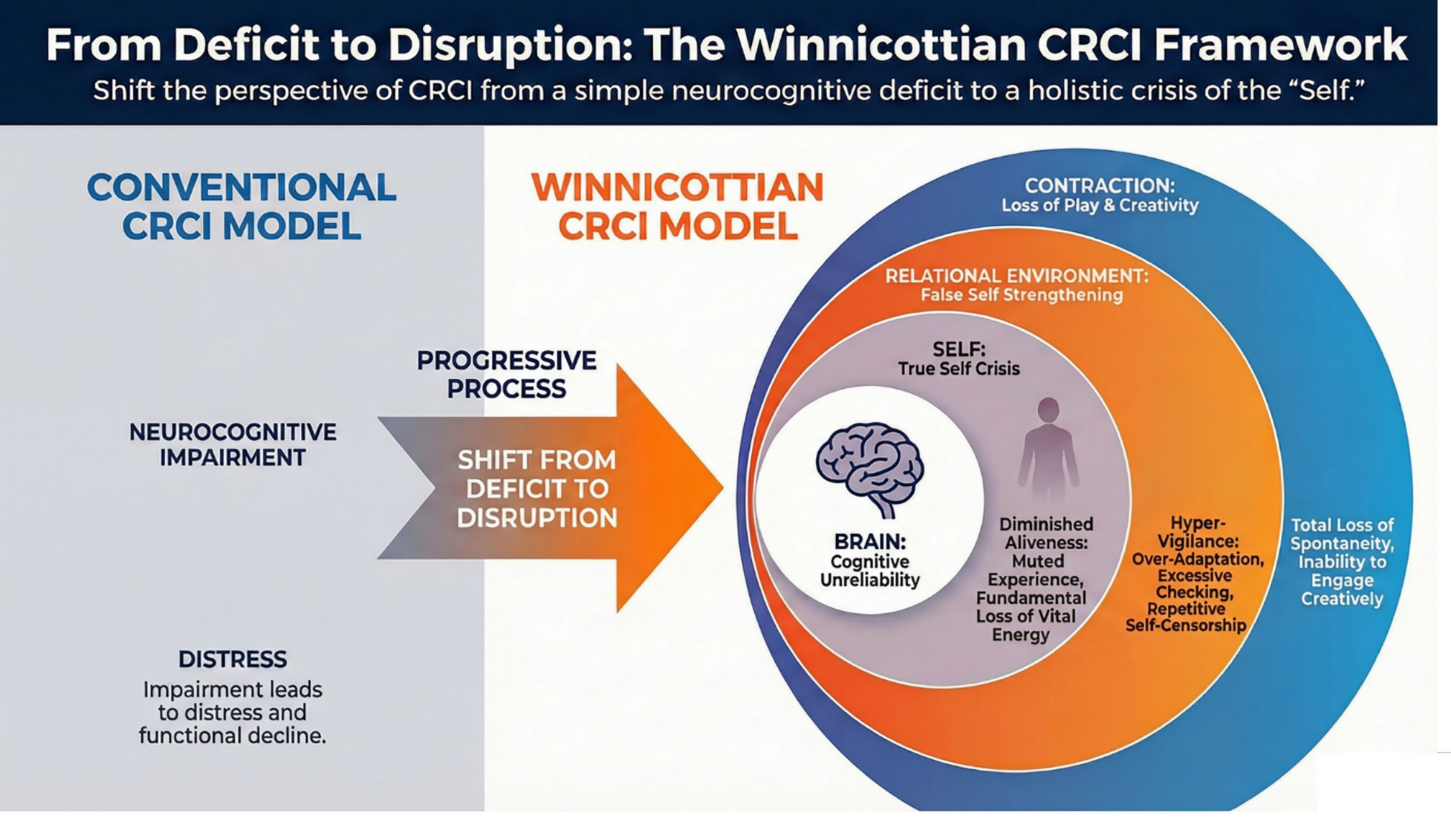
Winnicottian model of self-experience in cancer-related cognitive impairment. This figure contrasts the conventional neurocognitive deficit model of chemotherapy-related cognitive impairment (CRCI) with a Winnicottian reconceptualization. On the left, CRCI is depicted as a linear sequence in which neurocognitive impairment leads to distress and functional decline. On the right, the Winnicottian framework situates CRCI as a progressive disruption of selfhood: cognitive unreliability destabilizes the True Self, intensifies False Self functioning, and contracts the potential space of play and creativity. Concentric layers illustrate how brain-level dysfunction reverberates through self-experience and relational environment, producing diminished aliveness, hyper-vigilance, and loss of spontaneity. This model reframes CRCI not merely as a cognitive impairment but as a crisis of the self, emphasizing the need for therapeutic holding, and restoration of play.

Taken together, these constructs are not proposed as direct psychometric entities, but as clinically observable dimensions that can be triangulated through narrative, relational, and functional measures. Such an approach preserves the depth of the psychoanalytic framework while rendering it accessible to empirical inquiry and interdisciplinary dialogue within psycho-oncology. Figure 1 illustrates a Winnicottian model of self-experience in cancer-related cognitive impairment, emphasizing the relational organization of cognitive change.

#### 2.3.3 Conceptual implications for clinical understanding

Chemotherapy-related cognitive impairment is increasingly recognized not only as a neurocognitive sequela of cancer and its treatments, but also as a profound disruption of subjective self-experience, continuity, and agency. Conventional approaches to CRCI have largely emphasized cognitive rehabilitation or compensatory strategies aimed at optimizing cognitive performance. While such approaches may yield functional benefits, they often insufficiently address the existential distress, shame, and fragmentation of identity reported by survivors, particularly those with high premorbid cognitive centrality.

To address this gap, the present framework situates CRCI within a Winnicottian developmental and relational understanding. From this perspective, cognitive unreliability is not approached as a defect requiring correction, but as a condition that challenges the individual’s capacity to sustain a coherent and trustworthy sense of self within interpersonal and environmental contexts. The central emphasis thus shifts from normalization of function to the restoration of *livability*—the capacity to inhabit one’s current cognitive and emotional reality without excessive defensive self-organization.

Grounded in Winnicott’s concepts of holding, and potential space, this framework highlights how disruptions in self-continuity may be exacerbated or alleviated by the reliability of the surrounding environment. Survivors with CRCI frequently describe heightened vigilance, self-surveillance, and compensatory over-control as dominant modes of everyday functioning. Within a Winnicottian reading, these patterns can be understood as adaptive responses to perceived internal and environmental unreliability, rather than as maladaptive coping or resistance. When such adaptations persist beyond the context of acute threat, however, they may contribute to emotional exhaustion, constriction of spontaneity, and erosion of experiential vitality.

Accordingly, from a Winnicottian perspective, the availability of a reliable holding environment emerges as a central condition under which cognitive lapses, confusion, and emotional dysregulation can be experienced without corrective pressure or evaluative threat. In such conditions, vulnerability does not necessitate concealment or over-adaptation, allowing experiences of limitation to be borne without collapse of self-coherence.

##### 2.3.3.1 Conceptual dimensions of a holding-oriented psychotherapeutic understanding of CRCI

Table 2 summarizes key conceptual dimensions of a holding-oriented understanding of CRCI, illustrating how Winnicottian principles of holding, and play may inform the interpretation of clinical phenomena associated with cognitive unreliability. These dimensions are not intended to specify techniques, procedures, or sequences of care, but to clarify the relational and experiential conditions that support psychological continuity under conditions of altered cognitive capacity.

##### 2.3.3.2 Clinical implications

Viewed through this conceptual lens, CRCI is reframed as a relational and experiential condition rather than solely a functional deficit. Attention is therefore directed toward the quality of the environment in which survivors attempt to live with cognitive change, including the degree to which vulnerability is tolerated, dependency is legitimized, and spontaneity is permitted. Within such environments, compensatory strategies and cognitive supports may be experienced not as markers of deficiency, but as contextually appropriate adaptations that preserve dignity and self-coherence. This framework offers a set of interpretive priorities that may inform interdisciplinary dialogue and future empirical exploration at the intersection of psycho-oncology and survivorship research. Supplementary materials: Clinical Focus 1–3 and Supplementary Table S1 provide illustrative clinical language examples that clarify how the proposed Winnicottian framework may be communicated in therapeutic contexts.

#### 2.3.4 The holding environment, and play elaboration

##### 2.3.4.1 Holding: containing anxiety without collapse

Winnicott’s concept of the holding environment is central to understanding how recovery from CRCI may be psychologically possible. In psycho-oncology, relational and psychosocial containment has likewise been emphasized as a core condition for supporting patients facing existential threat, uncertainty, and identity disruption in the context of cancer (Grassi, 2020). Holding refers not merely to emotional support, but to an environment—relational, temporal, and symbolic—that can contain anxiety without requiring immediate adaptation or defense.

For patients with CRCI, the experience of cognitive unreliability often evokes intense anxiety, shame, and fear of exposure. Qualitative studies describe how even minor lapses are experienced as catastrophic, as though each failure confirms a deeper personal defect (Henderson et al., 2019). In this context, clinical interactions that emphasize reassurance, positive thinking, or efficiency may inadvertently function as environmental failures, mirroring earlier experiences in which spontaneity could not be safely expressed. The clinical implications of this Winnicottian framework can be further specified through three interrelated dimensions—holding, and play— which are summarized in Table 2 and further illustrated in Supplementary Table S1. A Winnicottian holding environment for CRCI is characterized by several key features. First, it allows patients to articulate experiences of loss, anger, and estrangement without pressure to reinterpret these feelings as maladaptive cognitions. Statements such as *“I’m not who I used to be”* are not challenged or reframed prematurely, but received as meaningful expressions of altered self-experience.

Second, holding involves tolerance for regression and dependency. CRCI often reactivates earlier developmental vulnerabilities related to competence, autonomy, and approval. Patients may require repetition, slower pacing, written summaries, or assistance with tasks they previously managed independently. When these needs are met without humiliation or impatience, the environment communicates that the self remains worthy of care despite diminished capacity. Third, holding protects the patient from excessive environmental demand. This may include advocating for workplace accommodations, structuring clinical encounters to reduce cognitive overload, or explicitly legitimizing the patient’s right to function “below previous standards” without moral failure. Such protections reduce the need for False Self adaptation and conserve psychic energy for integration.

Within a sufficiently holding environment, patients can begin to experience that their confusion and limitation do not annihilate relationships. This realization is foundational for the re-emergence of the True Self.

##### 2.3.4.2 Play: reopening potential space

The distinctive contribution of a Winnicottian framework to CRCI lies in its emphasis on *play*. For Winnicott, play is not merely leisure or recreation, but the experiential foundation of creativity, meaning, and authentic living (Winnicott, 1968). It occurs within the *potential space* between inner reality and external demands, where actions are neither fully dictated by necessity nor entirely disconnected from reality.

CRCI often severely constricts this potential space. As cognitive reliability diminishes, patients may become preoccupied with avoiding errors and maintaining appearances, a pattern consistently described in qualitative studies of CRCI and cancer survivorship (Henderson et al., 2019). Activities once undertaken for pleasure or curiosity are abandoned in favor of tightly controlled routines. Life becomes organized around risk minimization rather than exploration.

Work aimed at restoring play does not require formal play therapy or artistic production, though these may be helpful. Rather, it involves helping patients rediscover domains of experience in which performance is not the primary metric of value. Gardening, listening to music, informal writing, cooking without strict recipes, or gentle experimentation with new interests can all function as sites of play.

What distinguishes play from distraction is the presence of subjective aliveness. In play, patients momentarily experience themselves as agents rather than managers of deficiency. Even fleeting moments of enjoyment or absorption can signal that the True Self remains accessible.

Importantly, play should not be prescribed as a therapeutic task with expected outcomes. When play becomes instrumentalized, it risks being absorbed into False Self functioning. Instead, clinicians may support play indirectly by legitimizing non-productive time, reducing self-criticism, and modeling curiosity rather than evaluation.

In sum, through a Winnicottian lens, CRCI emerges not merely as a cognitive disorder but as a relational and developmental crisis that challenges the continuity of the True Self. A conceptual framework grounded in holding, and play offers pathways for understanding recovery that extend beyond functional remediation, pointing toward the reclamation of authenticity, creativity, and a renewed sense of being.

## 3 Discussion

### 3.1 Theoretical integration: chemotherapy-related cognitive impairment as a crisis of the self

The present paper proposed a psychoanalytic reconceptualization of CRCI grounded in Donald W. Winnicott’s theory of the self and environment. In this framework, biological findings are not treated as direct explanations of subjective experience, but as *background conditions* that shape the reliability of the self–environment regulatory system within which self-experience unfolds. Biological processes should be understood not as external or contextual factors, but as integral to the organization of self-experience. From a biopsychosocial perspective, brain function, affective regulation, and relational context are mutually constitutive. Neuroinflammatory and metabolic disruptions impair synaptic plasticity in hippocampal and prefrontal–limbic circuits, undermining cognitive reliability and destabilizing affective regulation through amygdala disinhibition and HPA axis activation (Ulrich-Lai and Herman, 2009; Yirmiya and Goshen, 2011; McEwen and Morrison, 2013; Hodes et al., 2015; Lim et al., 2016; Lee et al., 2019; Moodie et al., 2020; Sun et al., 2026). As affective predictability decreases, relational attunement and co-regulation become increasingly difficult to sustain (Feldman, 2007), thereby limiting the availability of holding. In turn, this threatens the continuity of the True Self, culminating in experiences of psychological unreality and self-fragmentation (Winnicott, 1960b).

As shown in Figure 1, the Winnicottian CRCI framework emphasizes the relational and experiential dimensions of impairment by situating cognitive unreliability within the continuity of self and environment. Neurobiological alterations, including metabolic changes and neuroinflammation, are understood as conditions that destabilize cognitive predictability and internal reliability rather than as direct determinants of subjective meaning. In this sense, such disruptions undermine the continuity of being. CRCI may, in a subset of patients, be more accurately understood as a crisis of the True Self, particularly in individuals with high premorbid cognitive centrality and a perceived disruption of self-continuity.

As summarized in Table 1, the Winnicottian framework shifts the locus of CRCI from functional cognitive deficit to a disturbance in self-continuity, thereby reframing emotional distress as constitutive rather than secondary. This formulation bridges an important gap between neurobiological and psychosocial models. By contrast, a Winnicottian perspective situates cognition within the broader organization of the self. Cognitive capacities are integral to how individuals experience agency and coherence; when they become unreliable, what is destabilized is not only performance but the implicit trust in one’s own being, marking a developmental rupture in some patients.

This section integrates neurobiological and psychoanalytic perspectives to reconceptualize CRCI as a crisis of the self. While CRCI has been emphasized as a source of disruption to self-experience, it is important to situate it within the broader context of cancer-related existential distress. A cancer diagnosis and its treatment are inherently disruptive experiences that can profoundly affect identity, self-perception, and continuity of being, independent of cognitive impairment. In this context, CRCI should not be conceptualized as an isolated or primary source of disruption, but rather as a distinct and interacting factor that may intensify or qualitatively shape disturbances in self-experience within an already destabilized experiential field. Accordingly, CRCI is best understood as a mechanism that operates within, and may amplify, an already destabilized self-experience.

**Figure 2:**
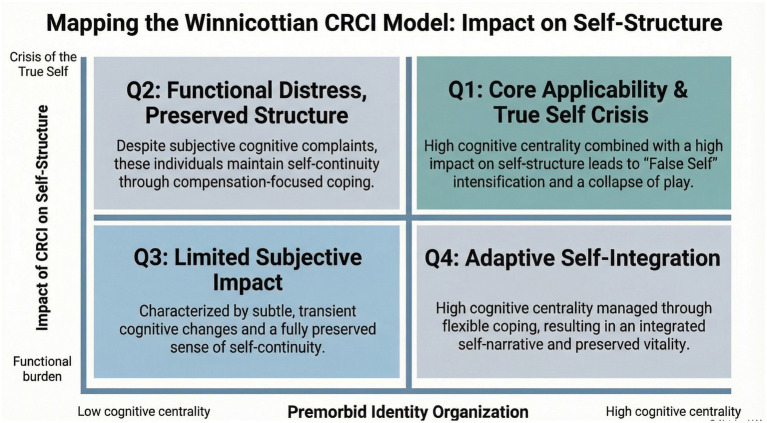
Conceptual quadrants of self-experience based on spontaneity and defensive adaptation. The figure illustrates how the proposed framework is most applicable to survivors with high premorbid cognitive centrality who experience CRCI as a disruption of self-continuity (Q1), while also distinguishing trajectories characterized by functional distress without self-structural disruption (Q2), limited subjective impact (Q3), and adaptive self-integration (Q4). Quadrant distinctions are intended as heuristic, cross-sectional formulations to guide conceptual applicability rather than as diagnostic, staging, or clinical triage categories. These configurations are dynamic, with individuals able to move between them over time. The quadrants are intended to guide conceptual fit rather than to classify patients. Comparative features distinguishing Q1 from Q2 are summarized in Supplementary Table S2.

Figure 2 presents a conceptual mapping of self-experience based on the dynamic interplay between spontaneity and defensive adaptation. A detailed description of Figure 2 is provided in the Supplementary materials. Consistent with recurring descriptions in the qualitative CRCI literature, disruptions in cognitive reliability are frequently experienced not merely as functional loss, but as threats to self-continuity and identity. While psychosocial and phenomenological models provide detailed descriptions of distress and altered self-experience in CRCI, they largely remain at a descriptive level. In contrast, the Winnicottian framework specifies a process-level mechanism in which cognitive unreliability destabilizes self-continuity, precipitates defensive self-organization (i.e., False Self functioning), and constricts the capacity for play within a relational context. This constitutes an incremental theoretical contribution by locating these disruptions within a relational and defensive organization of the self.

### 3.2 Reconceptualizing CRCI: from neurocognitive deficit to altered self-experience

Prevailing models of CRCI conceptualize the condition primarily as a disruption of discrete cognitive functions—such as attention, memory, and processing speed—attributed to neurotoxic treatment effects, inflammation, or altered neural connectivity, with subjective complaints interpreted as indicators of neurocognitive inefficiency or depleted self-regulatory resources. While these models, including compensatory and self-regulatory perspectives, have substantially advanced understanding of CRCI, they largely frame the phenomenon as a problem of cognitive performance. The present theoretical analysis proposes that this focus may obscure a central dimension of CRCI as lived by young adult cancer survivors, namely a qualitative transformation in how the self is experienced and organized. As outlined earlier, cognitive unreliability may promote reliance on False Self functioning, preserving functioning through heightened control while contributing to reduced spontaneity and self-estrangement. Applying Winnicott’s psychoanalytic framework allows CRCI to be reinterpreted not simply as impaired cognition, but as a qualitative shift in self-experience. Across the re-analyzed case narratives, CRCI manifested as diminished spontaneity, loss of experiential aliveness, and erosion of play capacity—hallmarks of what Winnicott described as inhibition of the *True Self* and dominance of the *False Self*. In this view, CRCI does not merely slow thinking; it reorganizes the way individuals relate to themselves and the world. Survivors may remain cognitively functional yet experience life as flattened, effortful, or excessively monitored. Importantly, this reorganization is not inherently pathological; rather, it represents a defensive adaptation that emerges under conditions of sustained threat.

During cancer treatment, such adaptations may be necessary for psychological survival. However, when these defensive structures persist after treatment completion, they may constrain recovery by preventing the re-emergence of spontaneity, creativity, and play.

A key contribution of Winnicott’s theory lies in the concept of the *holding environment*. During active cancer treatment, patients are embedded in a highly structured external holding environment comprising medical care, routines, surveillance, and relational containment. This environment may temporarily compensate for disruptions in internal stability.

Our analysis suggests that CRCI may persist when this external holding is withdrawn without being sufficiently internalized. In such cases, the individual remains psychologically organized around vigilance and self-protection, even in the absence of ongoing threat. Cognitive symptoms, therefore, may reflect not unresolved neural damage alone, but a failure of internal holding, resulting in continued reliance on external structure and control.

This interpretation helps explain why CRCI often persists despite objective cognitive recovery and why conventional cognitive rehabilitation may yield limited subjective benefit.

### 3.3 Clinical implications: reframing recovery

This reconceptualization has important implications for clinical practice. First, it challenges the implicit goal of many CRCI interventions: the restoration of premorbid cognitive functioning. While such recovery may be desirable, framing it as the primary therapeutic aim risks reinforcing False Self dynamics by positioning the patient’s current self as deficient or provisional. Qualitative studies of CRCI consistently indicate that patients’ distress often arises not from isolated cognitive failures, but from sustained efforts to preserve coherence, competence, and credibility in the face of perceived internal unreliability.

From a Winnicottian standpoint, recovery is not synonymous with returning to a previous state, but with the re-establishment of conditions under which the True Self can exist and express itself, even within altered limits. Within this framework, recovery may be reconceptualized as a shift from optimizing performance to facilitating livability—the capacity to inhabit one’s current cognitive reality without pervasive self-alienation. Second, the emphasis on holding underscores the relational nature of recovery. Cognitive rehabilitation, psychoeducation, and compensatory strategies are most effective when delivered within an environment that legitimizes vulnerability and tolerates regression. When clinicians acknowledge cognitive limitation without urgency to fix it, patients are less compelled to hide, overcompensate, or withdraw. Third, the restoration of play emerges as a central therapeutic indicator. Play signals the reopening of potential space, where patients can experience themselves as alive rather than merely functional. Importantly, play should not be imposed as an intervention with measurable outcomes, but supported as a spontaneous byproduct of safety and acceptance. Its presence often marks a shift from survival-oriented adaptation to creative living, and thus serves as a clinically meaningful indicator of psychological recovery.

Moreover, given that the core of this dynamic lies in the ongoing movement between spontaneity and defensiveness, qualitative research approaches—including narrative interviews, longitudinal case analyses, and therapist-rated process indicators focusing on spontaneity, symbolic expression, and relational flexibility—are particularly well suited to capturing changes in self-organization over time and to complementing patient self-report data. The Supplementary materials provide illustrative clinical language examples intended to clarify how the proposed conceptual framework might inform therapeutic communication in practice.

From a psycho-oncological perspective, these findings highlight the clinical importance of attending not only to cognitive functioning but also to the relational context in which survivors make sense of cognitive change after treatment.

### 3.4 Limitations of the Winnicottian approach

Several limitations of the present conceptual framework must be acknowledged. First, this paper is primarily theoretical and illustrative in nature. While grounded in existing psycho-oncological and psychosomatic literature, it does not provide empirical data demonstrating that Winnicottian-informed interventions lead to superior outcomes compared to standard cognitive rehabilitation alone. Future research will be required to operationalize, or develop empirically tractable proxies for, constructs such as True Self functioning, holding environment, and play in ways amenable to systematic study. Second, Winnicott’s theory emerged from a specific psychoanalytic and cultural context, and its applicability across diverse populations requires careful consideration. Experiences of selfhood, autonomy, and relational dependence are shaped by cultural norms, socioeconomic conditions, and healthcare systems. The meaning of cognitive loss—and the forms of False Self adaptation it elicits—may differ substantially across contexts. Patient experiences of CRCI are heterogeneous. While some individuals may experience cognitive changes as a disruption of self-continuity, others may experience primarily functional difficulties or minimal subjective impact. The present framework is therefore intended to apply selectively, rather than as a generalized characterization of CRCI.

Third, not all patients with CRCI experience their cognitive changes as identity-threatening. Some individuals adopt a pragmatic stance toward impairment and do not report significant existential distress. It is important to note that such False Self adaptations are not inherently pathological; rather, they may initially function as adaptive strategies that become constraining only when rigidly maintained in the absence of sufficient environmental holding.

Finally, there is a risk that psychoanalytic formulations may be misunderstood as minimizing the biological reality of CRCI. This paper does not propose a replacement of neurobiological models, but a complementary framework that situates biological impairment within lived experience. Maintaining dialogue between these perspectives remains essential.

### 3.5 Directions for future research

Future research should adopt longitudinal and interdisciplinary approaches to clarify how CRCI evolves over time and how False Self adaptation develops in response to disruptions in holding and environmental reliability. Longitudinal designs capturing intra-individual change will be essential for distinguishing transient defensive responses from more enduring reorganizations of self-structure and for identifying conditions under which initially protective adaptations become maladaptive. Integrating PET-based markers of neuroinflammation with psychodynamic, qualitative, and phenomenological assessments may further elucidate the relationship between brain function, self-organization, and lived experience. In parallel, intervention studies incorporating holding-oriented psychotherapy alongside cognitive rehabilitation may help determine whether improvements in perceived holding are associated with broader gains in vitality, relational engagement, and quality of life.

## 4 Conclusion

This framework positions CRCI as a relational and experiential disruption rather than a purely cognitive deficit, suggesting that recovery depends not on compensatory adaptation alone but on the restoration of relational conditions that sustain authentic continuity of being. By foregrounding disruptions in holding, and play, CRCI is reframed as a disturbance of subjectivity, thereby extending clinical understanding beyond functional normalization and highlighting the importance of relational conditions through which survivors sustain a livable sense of self following treatment. Critically, this framework advances the field by specifying the relational and defensive mechanisms through which cognitive unreliability is transformed into a disruption of self-continuity.

## Data Availability

The data analyzed in this study is subject to the following licenses/restrictions: the data that support the findings of this study are available from the corresponding author upon reasonable request. Requests to access these datasets should be directed to Jin Su Kim, kjs@kirams.re.kr.
