# Supplementary material for: Chemotherapy-related cognitive impairment as a crisis of the self: a Winnicottian psycho-oncological perspective
Source: Front Psychol. 2026 May 14;17:1811745. doi: 10.3389/fpsyg.2026.1811745 (PMC13217401; doi:10.3389/fpsyg.2026.1811745)
Supplement: Supplementary file 1 [file Data_Sheet_1.docx]

**Supplementary material**

**Chemotherapy-Related Cognitive Impairment as a Crisis of the Self: A Winnicottian Psycho-Oncological Perspective**

Authors: Sun Mie Kang^1^, Jin Su Kim^2,3^ ^†^

Affiliations:

^1^Major in Psychoanalytic Counseling, Konkuk University, 120 Neungdong-ro, Gwangjin-gu, Seoul 05029, Republic of Korea

^2^Division of Applied RI, Korea Institute of Radiological and Medical Sciences (KIRAMS), 75 Nowon-ro, Nowon-gu, Seoul 01812, Republic of Korea

^3^Radiological and Medical Sciences, University of Science and Technology (UST), 75 Nowon-ro, Nowon-gu, Seoul 01812, Republic of Korea

Corresponding authors

†Jin Su Kim, PhD

Division of Applied RI, Korea Institute of Radiological and Medical Sciences (KIRAMS), 75 Nowon-gu, Seoul 01812, Korea

Tel: 82-2-970-1661,

Email: [kjs@kirams.re.kr](mailto:kjs@kirams.re.kr)

___________________________________________________________________________

*The following materials are provided as conceptual illustrations intended to clarify theoretical implications for clinical practice. They are not intended as a treatment manual, protocol, or prescriptive guide, and no claims of empirical efficacy are made.*

___________________________________________________________________________

**I. Conceptual Principles Underlying a Holding-Oriented Approach**

The proposed Winnicottian framework suggests that therapeutic work with CRCI survivors may unfold across several overlapping conceptual phases. These phases are not rigidly sequential and should be adapted to individual clinical contexts.

**Clinical Focus 1: Establishing a Holding Environment**

In many clinical contexts, particularly at points where trust is fragile, the therapeutic priority may become to establish predictability, safety, and a non-evaluative presence. The therapeutic environment is often characterized by a deliberately slow pace and simple structure, allowing the patient to experience being able to remain in the relationship before being asked to improve or perform. Cognitive lapses, fragmented speech, and repetitions are received without correction.

*Illustrative therapist language:*

*"Today, rather than starting with an evaluation, I'd like to hear what it was like for you to come here."*

*"We are not working to restore who you used to be. We are working so that you can live as you are now."*

**Clinical Focus 2: Recognizing Adaptive Patterns**

As the relationship stabilizes, attention may turn to patterns of over-adaptation that the patient employs to manage cognitive unreliability—such as excessive checking, vigilance, or avoidance of situations that might expose vulnerability. Within a Winnicottian frame, these are understood as protective strategies rather than pathology, and are named without interpretive pressure.

*Illustrative therapist language:*

*"It seems you're working very hard not to be wrong. That effort itself feels like something that once helped you survive."*

When shame or fear of exposure emerges, the therapist tolerates silence and refrains from premature reassurance or analysis of causes.

**Clinical Focus 3: Environmental Adaptation**

As therapy progresses, concrete environmental adjustments may support the patient's capacity to remain engaged without excessive self-monitoring. Such adaptations might include simplifying session structure, explicitly permitting repetition, or providing brief written summaries.

*Illustrative therapist language:*

*"You may ask the same question multiple times. This is not a memory test."*

**Ongoing Dimension: Play and Potential Space**

Throughout the process, opportunities may arise for the patient to experience moments of lightness, experimentation, or creative engagement—what Winnicott termed "play." These moments are not prescribed but recognized and supported when they emerge spontaneously.

**A shift in self-narrative may gradually occur:**

| Before | After | |
| --- | --- | --- |
| “I am broken” | **“I function differently”** |  |

*The therapist participates as a co-editor of emerging self-understanding rather than as an author of the patient’s narrative.*

As therapeutic work approaches moments of separation or transition, the emphasis remains on continuity rather than closure.

***Illustrative therapist language:***

*"If difficulties return, it does not mean you have collapsed."*

**II. Examples of Holding-Oriented Communication**

The following examples illustrate how the holding-oriented stance described in the main text might be communicated to patients. These are illustrative only and would be adapted to individual clinical contexts.

*Example of framing the therapeutic relationship:*

*"Repeating yourself is allowed. Disorganized thoughts are allowed. Here, relationships remain even without perfect performance."*

*Example of acknowledging ongoing vulnerability:*

*"Difficulties may return after our work together changes or concludes. This does not mean failure—it means you are living with something that continues."*

**Table S1.** Conceptual Dimensions of CRCI: Conventional vs. Winnicottian Interpretation

| **Conventional CRCI interpretation** | **Winnicottian Reconceptualization  (This Study)** |
| --- | --- |
| Slowed cognitive processing | Loss of experiential aliveness |
| Resource depletion | Defensive self-organization |
| Compensatory coping strategies | Structural reinforcement of the *False Self* |
| Cognitive avoidance | Collapse of play capacity |
| Self-regulatory depletion | Failure to internalize holding |

**Detailed Description of Figure 2**

Q1 represents the core applicability of the framework, reflecting a subset of patients, in which high premorbid cognitive centrality renders CRCI disruptive to self-continuity, with associated True Self disruption, intensified False Self functioning, and collapse of play. Q2–Q4 represent trajectories in which a Winnicottian depth formulation is neither necessary nor appropriate. In Q2, self-structure remains largely intact, and distress is primarily functional in nature; as such, standard psychoeducational or cognitive rehabilitation approaches are likely to be sufficient. In Q3, the subjective impact of cognitive change is minimal, and the application of a depth-oriented psychoanalytic framework would risk unnecessary pathologization. However, clinicians should be cautious not to interpret Q3 as indicating the absence of distress. A low-signal presentation may reflect unarticulated or defended distress rather than its absence, and warrants continued clinical attention rather than dismissal. In Q4, adaptive self-integration has already occurred, rendering therapeutic intervention aimed at restoring self-continuity largely redundant. These quadrants are not intended as diagnostic or classificatory categories, but as heuristic, cross-sectional formulations that describe patterns of self-organization at a given point in time. Although presented as discrete regions for conceptual clarity, they are inherently dynamic, and individuals may move between quadrants over time depending on changes in cognitive reliability, relational context, and defensive organization. Explicitly distinguishing these trajectories is essential not only for conceptual clarity, but also for preventing the overextension of the Winnicottian model beyond the clinical contexts in which it offers genuine explanatory and therapeutic value.

**Table S2.** Comparative Features of True Self Disruption and False Self Dominance Versus Preserved True Self Functioning

| **Domain** | **Q1: True Self Disruption / False Self Dominance** | **Q2: Preserved True Self / Functional Distress** |
| --- | --- | --- |

| **Core organization** | Disruption of self-continuity; defensive reorganization of the self | Self-continuity preserved; no dominant defensive reorganization |
| --- | --- | --- |
| **Subjective experience** | “I am no longer myself”; sense of alienation from self | “I am slower / forgetful”; frustration without identity threat |
| **Language** | Identity-level expressions; self-discontinuity | Function-level descriptions; performance concerns |
| **Behavior** | Compulsive self-monitoring, concealment of lapses, over-adaptation, rigid routines | Open use of compensatory strategies (notes, structure) without concealment |
| **Affect** | Constricted; prominent shame, fear of exposure | Flexible; frustration, fatigue without shame |
| **Relational style** | Compliant, over-organized, performative; defensive engagement | Flexible, non-defensive, open acknowledgment of limitations |
| **Response to disruption** | Rapid restoration of coherence; intolerance of disorganization | Tolerance of minor disruption; no urgency to restore coherence |
| **Defensive pattern** | False Self dominance; over-control and compliance | No dominant False Self organization; adaptive coping |
| **Play capacity** | Markedly reduced; diminished spontaneity, humor, symbolic exploration | Preserved; spontaneity, curiosity, and tolerance of ambiguity intact |
| **Clinical meaning** | Cognitive disruption experienced as threat to the self | Cognitive difficulty experienced as functional challenge |
